# A25 MEASURING THE OBSERVER (HAWTHORNE) EFFECT ON ADENOMA DETECTION RATES: A CASE-CONTROL RETROSPECTIVE STUDY

**DOI:** 10.1093/jcag/gwac036.025

**Published:** 2023-03-07

**Authors:** M Taghiakbari, D E Coman, M Takla, A N Barkun, M Frija-Gruman, M Bouin, S Bouchard, E Deslandres, S Sidani, D von Renteln

**Affiliations:** 1 University of Montreal; 2 University of Montreal Hospital Research Center (CRCHUM); 3 Division of Internal Medicine, University of Montreal Hospital Center (CHUM); 4 Division of Gastroenterology, McGill University Health Center, McGill University; 5 Division of Gastroenterology, University of Montreal Hospital Center (CHUM), Montreal, Canada

## Abstract

**Background:**

The effectiveness of colonoscopy screening to prevent colorectal cancer (CRC) is directly linked to its procedural quality. An independent observer (Hawthorne effect) can improve colonoscopy procedural quality metrics, including adenoma detection rate (ADR). However, the results of studies are limited or controversial.

**Purpose:**

We aimed to evaluate the colonoscopy quality metrics in a group of patients undergoing screening or diagnostic colonoscopies under stringent observer conditions.

**Method:**

In a single-center, case–control study, consecutive patients undergoing routine screening or diagnostic colonoscopy were prospectively enrolled. In the case group, all procedural steps and quality metrics were observed and documented, and the procedure was video recorded by an independent research assistant. In the control group, colonoscopies were performed without independent observation. Colonoscopy quality metrics such as polyp, adenoma, serrated lesions, and advanced adenoma detection rates (PDR, ADR, SLDR, AADR), the mean number of adenomas detected per patient (MAP), and the mean number of adenomas and serrated lesions detected per patient (MASP) were compared. The probabilities of increased quality metrics were evaluated through regression analyses weighted by the inversed probability of observation during the procedure.

**Result(s):**

We included a total of 687 patients (327 cases and 360 controls) in the final analyses. The case group had significantly higher PDRs (62.4% vs. 53.1%) and ADRs (39.4% vs. 28.3%) compared with the control group. The SLDR was also higher in the case group than in the control group, but the difference was not significant (7.3% vs. 4.4%; P = 0.14). The AADR was not significantly increased. After adjusting for potential confounders, the ADR and SLDR were 50% (odds ratio [OR] 1.51; 95%CI 1.05–2.17) and more than twofold (OR 2.17; 95%CI 1.05–4.47) more likely to be higher in the case group than in the control group. The MAP and MASP were significantly increased in the case group compared with the control group (P < 0.001). The regression analyses for both metrics demonstrated the direct and significant association between the Hawthorne effect and elevated MAP/MASP.

**Conclusion(s):**

The presence of an independent observer documenting colonoscopy quality metrics and video recording the colonoscopy resulted in a significant increase in ADR and other quality metrics. The Hawthorne effect should be considered an alternative strategy to advanced devices to improve colonoscopy quality in routine practice.

**Please acknowledge all funding agencies by checking the applicable boxes below:**

None

**Disclosure of Interest:**

M. Taghiakbari: None Declared, D. Coman: None Declared, M. Takla: None Declared, A. N. Barkun: None Declared, M. Frija-Gruman: None Declared, M. Bouin: None Declared, S. Bouchard: None Declared, E. Deslandres: None Declared, S. Sidani: None Declared, D. von Renteln Grant / Research support from: ERBE, Ventage, Pendopharm, and Pentax, Consultant of: Boston Scientific and Pendopharm

